# Footedness predicts escape performance in a passerine bird

**DOI:** 10.1002/ece3.6193

**Published:** 2020-04-16

**Authors:** Gaoyang Yu, Jinxin Guo, Wenqian Xie, Jun Wang, Yichen Wu, Jinggang Zhang, Jiliang Xu, Jianqiang Li

**Affiliations:** ^1^ School of Ecology and Nature Conservation Beijing Forestry University Beijing China; ^2^ Ministry of Education Key Laboratory for Biodiversity Sciences and Ecological Engineering College of Life Sciences Beijing Normal University Beijing China

**Keywords:** behavioral lateralization, escape performance, exploration, feeding efficiency, footedness, yellow‐bellied tit

## Abstract

Behavioral lateralization, which is associated with the functional lateralization of the two brain hemispheres, commonly exists in animals and can provide an individual with benefits such as enhanced cognition and dual tasking. Lateral bias in limb use, as a type of behavioral lateralization, occur in many species, but the reasons for the coexistence of left‐ and right‐biased individuals in a population remain poorly understood. We examined the footedness of male yellow‐bellied tits (*Pardaliparus venustulus*) when they used feet to clamp mealworms against a perch, and tested its association with other fitness‐related behavioral traits (i.e., feeding efficiency, exploration tendency, and escape performance). We expected differently footed individuals to have respective advantages in these behaviors and thereby coexist (“respective advantage” hypothesis). We found their footedness repeatable, and there was no population‐level bias. While no associations of feeding efficiency and exploration tendency with footedness were detected, the right‐footed individuals were found to be harder to catch than the other individuals. Future studies need to investigate the reasons for the right‐footed individuals' superior escape performance. Moreover, the escape advantage for being right‐footed and the lack of population‐level bias in footedness in male yellow‐bellied tits suggest that the benefits related to left footedness also remain to be explored.

## Graphical abstract

Behavioral lateralization, such as handedness and footedness, commonly exists in animals, but the benefits associated with differently lateralized individuals remain poorly understood. In this article, feeding efficiency, exploration performance, and escape performance of left‐, not‐ and right‐footed yellow‐bellied tits were tested. Right‐footed individuals were found to perform better in escaping, and such a phenomenon is worthy to be investigated in more species.

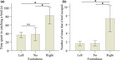

## 1 INTRODUCTION

The preferential use of left or right side of the body in behaviors widely exists in vertebrates (Csermely & Regolin, 2013; Rogers, 2002; Ströckens, Güntürkün, & Ocklenburg, 2013) and invertebrates (Frasnelli, 2013; Frasnelli, Vallortigara, & Rogers, 2012; Niven & Bell, 2018), occurring at the levels of both an individual organism and a population (Frasnelli & Vallortigara, 2018; Rogers, 2002; Rogers & Andrew, 2002). Behavioral lateralization has been shown to be heritable (Bisazza, Facchin, & Vallortigara, 2000; Brown, Western, & Braithwaite, 2007; Lien, Chen, Hsiao, & Tsuang, 2015) and is thought to be associated with the functional specialization of the two brain hemispheres, which receive afferents contralaterally (i.e., to the opposite side of body) and control the motor and sensory functions contralaterally (Frasnelli, 2013; Rogers, 2009; Rogers & Vallortigara, 2017). When one side of the brain is more dominant in processing certain stimuli, predominant uses of the contralateral side of the body may therefore occur (Rogers, 2009, 2014).

Behavioral lateralization exists in various contexts. During foraging, for example, it is often reported that the response to prey usually involves the use of the right eye that is controlled by the left hemisphere (e.g., Bonati, Csermely, & Romani, 2008; Robins, Chen, Beazley, & Dunlop, 2005; Robins & Rogers, 2004). On the other hand, the left eye, controlled by the right hemisphere, is usually used for threat detection in risky scenarios (e.g., Koboroff, Kaplan, & Rogers, 2008; Shibasaki, Nagumo, & Koda, 2014). These preferential uses of one side of the body and the differential specialization of the hemispheres can benefit an individual through enhanced ability to perform two different tasks simultaneously (Rogers, 2000; Rogers & Vallortigara, 2017). For example, it has been found that the lateralized domestic chicks (*Gallus domesticus*) have a better ability to find food while staying vigilant for predators compared to nonlateralized ones (Rogers, Zucca, & Vallortigara, 2004). Similarly, strong‐lateralized common marmosets (*Callithrix jacchus*) also show better ability to perform foraging and predator detection simultaneously than weak‐lateralized ones (Piddington & Rogers, 2013). It is now known that in vertebrates the right hemisphere is more likely to be involved in attending to novel stimuli (e.g., predation risk), expressing intense emotions, controlling social behavior, processing spatial information, and making decisions, while the left hemisphere is more specialized in focusing attention to perform learned tasks, following rules and categorizing stimuli (MacNeilage, Rogers, & Vallortigara, 2009; Rogers, 2002; Rogers & Vallortigara, 2017).

Individuals in a population may exhibit different lateral biases in the same behavior. For example, in species such as northern tree shrew (*Tupaia belangeri*; Maille et al., 2013) and capuchin monkeys (*Cebus paella*; Spinozzi, Truppa, & Laganà, 2004), some individuals prefer to use their left limb, while the others prefer the right limb when grasping. This phenomenon, however, presents a puzzle about why the behavior of a population is not selected to bias toward the same side during evolution, given the advantage of one side of the hemisphere in performing certain behaviors over the other as mentioned above. One way for differently lateralized individuals to coexist is that they have respective advantages under different circumstances (hereafter “respective advantage” hypothesis), with the advantage at one context paying off the costs at another (Chivers et al., 2017). The handedness of humans illustrates the situation: While left handers have been found to suffer certain costs compared with right‐handers, such as smaller body size and reduced longevity (reviewed in Llaurens, Raymond, & Faurie, 2009), they are advantaged in contests with conspecifics (Raymond, Pontier, Dufour, & Møller, 1996) and have better leadership (Mukherjee, 2017), which may balance the disadvantage.

However, the limb use in taxa other than humans and nonhuman primates remains less investigated and examination of whether the individuals with different limb use have different advantages and thereby coexist in a population is even rarer. In birds, species such as parrots (Brown & Magat, 2011; Rogers & Workman, 1993), crows (Izawa, Kusayama, & Watanabe, 2005) and tits (Gibb & Hartley, 1957; Yince, 1964) often use their feet to hold objects like food items, providing opportunities to investigate their footedness. Moreover, since lateral bias in limb use is likely a reflection of a general dominant role of one hemisphere over the other (Gordon & Rogers, 2015; Rogers, 2009), footedness in birds can be suitable subjects to test the respective advantage hypothesis for the coexistence of differently lateralized individuals in a population. This is because an individual's performance in other behaviors, which may be associated with the dominance status of either of the brain hemispheres, can be readily predicted based on the general dominance of the hemisphere reflected by its footedness and can be tested, an area of which has rarely been investigated in wild animals (Braccini & Caine, 2009).

In this study, we examined whether footedness can predict an individual's performance in other behaviors and whether individuals with different footedness have respective advantages to coexist in a population in yellow‐bellied tits (*Pardaliparus venustulus*), a bird species in the Paridae family. We investigated the association of footedness with individual performance in three behavioral traits that potentially affect an individual's fitness: the efficiency in feeding, exploration tendency, and escape performance from potential predation. We expected individuals of different footedness may have respective advantages in different behaviors or they perform equally well in these behaviors.

With respect to feeding efficiency, an individual may have an advantage if it eats faster, for example, when there are predators or conspecific competitors (Lima, 1985; Webster, 2004). Previous studies of behavioral laterality have found a correlation between the uses of the left hemisphere and enhanced foraging ability. For example, in both domestic chicks (Mench & Andrew, 1986) and pigeons (*Columba livia*; Güntürkün & Kesch, 1987), an individual has significantly higher accuracy in discriminating grains from pebbles when using the right eye than the left eye. Similarly, in wild black‐winged stilts (*Himantopus himantopus*), pecks with right eye detection succeed more often than pecks with left eye detection during foraging (Ventolini et al., 2005). Therefore, it is worthy to examine whether an individual's foraging efficiency is correlated with its dominant hemisphere reflected by its footedness. Here, we predicted the right‐footed yellow‐bellied tits, which are likely to have a dominant left hemisphere, to have a better feeding efficiency (i.e., to eat faster) than left‐footed yellow‐bellied tits.

For exploration behavior, it is a personality trait having been found to correlate with an individual's various aspects of life such as territory quality, dispersal distance, and survival (Dingemanse & Réale, 2005; Réale, Reader, Sol, McDougall, & Dingemanse, 2007). Regarding the relationship between exploration and behavioral lateralization, it has been found that common wall lizards (*Podarcis muralis*) preferentially use their left eye (thus the right hemisphere) to view the environment (Bonati & Csermely, 2011; Csermely, Bonati, Lopez, & Martín, 2011). Moreover, when exploring a new environment, the lizards using their left eye only react faster and more efficiently than those using the right eye only, suggesting that processing spatial stimuli mediated by the right hemisphere/left eye can provide an advantage (Bonati, Csermely, & Sovrano, 2013). On the other hand, in both common marmosets (Cameron & Rogers, 1999; Gordon & Rogers, 2015) and Geoffroy's marmosets (*Callithrix geoffroyi*; Braccini & Caine, 2009), it has been found that the left‐handed individuals are less likely than right‐handed individuals to inspect new objects, possibly because right hemisphere is associated with the expression of fear. Therefore, the left‐footed yellow‐bellied tits may be predicted to be more explorative for the right hemisphere's advantage in processing spatial stimuli compared to the right‐footed ones. Alternatively, they may be less explorative because of the association of the right hemisphere with fear expression.

Animals living in the wild often face kinds of risks and an individual's response to predation risks reflect its performance in avoiding predation. In this regard, the right hemisphere has been found to play an important role in controlling an individual's response to threats. For example, it has been found that stripe‐faced dunnarts (*Sminthopsis macroura*) are more likely to respond when using their left eye to detect a simulated predator (Lippolis, Westman, McAllan, & Rogers, 2005). Likewise, Australian magpies (*Gymnorhina tibicen*) that use their left eye more than their right eye produce stronger responses to risks (Hoffman, Robakiewicz, Tuttle, & Rogers, 2006). Moreover, as the right hemisphere is advantaged in processing spatial information (Rogers, 2002; Rogers & Vallortigara, 2017), an individual with a dominant right hemisphere may also be expected to perform better in fleeing and avoiding being caught when it is chased by a predator in a limited space. Hence, we predicted left‐footed yellow‐bellied tits to be better in escaping than right‐footed conspecifics.

Taken together, based on the specialized advantages of the two brain hemispheres about the above behaviors, we predicted that right‐footed (left hemisphere dominant) individuals should eat faster, but have poorer escape performance than left‐footed individuals, and in context of exploring a novel environment, they can be either more explorative or less explorative.

## 2 MATERIALS AND METHODS

### 2.1 Study species and general experimental procedures

Yellow‐bellied tits are endemic to central and eastern China (Zheng, 2017). The species weighs 9–12.5 g and has a total length of 10–11 cm. Males differ from females in plumage in that females are generally duller with a distinguishable olive‐green upper body, while males are brighter and have a blackhead contrasting with their white ear patch. The plumage of juveniles is similar to that of females but is sexually separable at an early age: within 1 year after being born, young males already resemble adult males but with distinguishable pale yellow chin and throat, and dull yellowish side of the neck. The birds are usually present in pairs during the breeding season, but in the nonbreeding season they often form large flocks. The diet of this species includes small invertebrates and seeds (Gosler & Clement, 2007). While feeding, yellow‐bellied tits usually use feet to clamp food items to a perch and then tear or hammer at them.

The experiments were conducted at the zoological laboratory of Beijing Forestry University from May to November 2015. Yellow‐bellied tits have a strong male‐biased population sex ratio (J. Li, unpublished data), so the males were studied for being relatively easy to catch. A total of 36 male yellow‐bellied tits caught with mist nets in suburban montane habitats of Beijing were involved, including six individuals caught in May and the remaining 30 caught in October. Among these, 26 were first‐year males and the other 10 were at least at their second year of age (hereafter, adults), as indicated by their plumage. To optimize experimental procedures, pilot studies were performed with the six individuals caught in May (all were adults); these six individuals were therefore not involved in the formal tests, reducing the sample size of the formal tests to 30 (26 first‐year individuals and four adults).

Except for during tests, yellow‐bellied tits were raised in cages of 50 cm long × 35 cm wide × 35 cm high, with five to seven individuals each. Birds were provided with water and commercial bird foods with additional silkworms, mealworms, crickets, and egg yolk as a daily diet. Before the formal tests, all birds were allowed to get habituated to the caged condition for at least 3 days. Formal tests were conducted in a sequence of footedness, exploration tendency, and escape performance. All birds were released after the tests in early November.

### 2.2 Test for footedness

Each bird's footedness was tested by individually putting them in a small cage (27 cm long × 13 cm wide × 20 cm high). To avoid mutual interaction between birds, the cages were visually isolated between each other by placing each cage in a box with opaque walls. After being transported into the experimental cage, each bird was given a habituation period of 1.5 hr. During this period, the birds were provided with water but no food, the purpose of which was to increase their hunger level to prepare for the footedness tests. After the habituation, 10 mealworms of similar size and color were delivered into the feeder in the cage through a hole on the sidewall of the box (the tested bird was hidden from human manipulation). The feeding behaviors of the bird in the following 15 min were recorded by a video camera set aside. From the videos, we recorded the foot that a bird used to grasp each mealworm for the first time, the foot used to clamp the mealworm against the perch when finishing eating it, the switches between left foot and right foot while eating, and the total time spent on each foot while eating each mealworm.

To investigate whether an individual's footedness was repeatable over time, a subgroup of the individuals (*N* = 21; 17 first‐year individuals and four adults) were tested for footedness repeatedly on the 4th, 7th, 14th, 21st, and 28th day after their first tests. Because of the failure to film the feeding process of a few birds for technical problems of the cameras and that some individuals directly swallowed the mealworms or did not feed during the tests, the final sample sizes varied between the tests on different days from 14 to 19 (see Table S1).

We used the laterality index, LI = (uses of the right foot − uses of the left foot)/(uses of the right foot + uses of the left foot; Bisazza, Pignatti, & Vallortigara, 1997) to describe both the direction and the level of laterality for footedness of each individual. LI varies continuously from −1 for completely left‐footed to +1 for completely right‐footed. When calculating LI, the cases that a bird used both feet for clamping the mealworms were excluded, which represented a small proportion of the total number of foot use (averagely 2.0% across individuals).

According to the behaviors recorded in the video, we calculated four different indexes of LI: (a) LI of total time spent in using a foot (LITT, reflecting a bird's preference for using a given foot to clamp the mealworm against the perch during the whole process); and (b) LI of total number of times of using a foot (LITN, similar to LITT, but calculated with the total number of times of using a foot, instead of the total time, during the whole process); (c) LI of first‐used foot (LIFF, reflecting the tendency of using a given foot to grasp food for the first time by a bird); (d) LI of last‐used foot (LILF, reflecting the tendency of using a given foot to clamp the mealworm against the perch when a bird was finishing eating each mealworm). The four indexes were correlated with each other in any of the six tests of footedness repeatability (Spearman correlations: *r* =0.661–.966, *N* = 14–19, all *p* < .01; see Table S1 for details).

### 2.3 Test for feeding efficiency

During the footedness tests, the time that a bird spent in eating a mealworm was recorded and was used for the analysis of feeding efficiency. Our data showed signs of decreasing time spent in eating a mealworm with more tests that birds experienced, which possibly reflected the birds' adaption to eat mealworms. Therefore, we used only the data of the first round of repeatability tests for the feeding efficiency analysis for the birds that had experienced repeated footedness tests. This treatment allowed us to include the data from birds that were not involved in the repeatability test. During three birds' tests, the mealworms (*N* = 5) were dropped onto the cage floor and the birds did not pick up them to continue feeding; these cases were removed from the analyses. Besides, three birds' data were not available due to technical problems of the cameras, so the feeding efficiency was analyzed for 27 yellow‐bellied tits (23 first‐year individuals and four adults) with 4–10 mealworms eaten by each bird.

### 2.4 Test for exploration tendency

**Figure 1:**
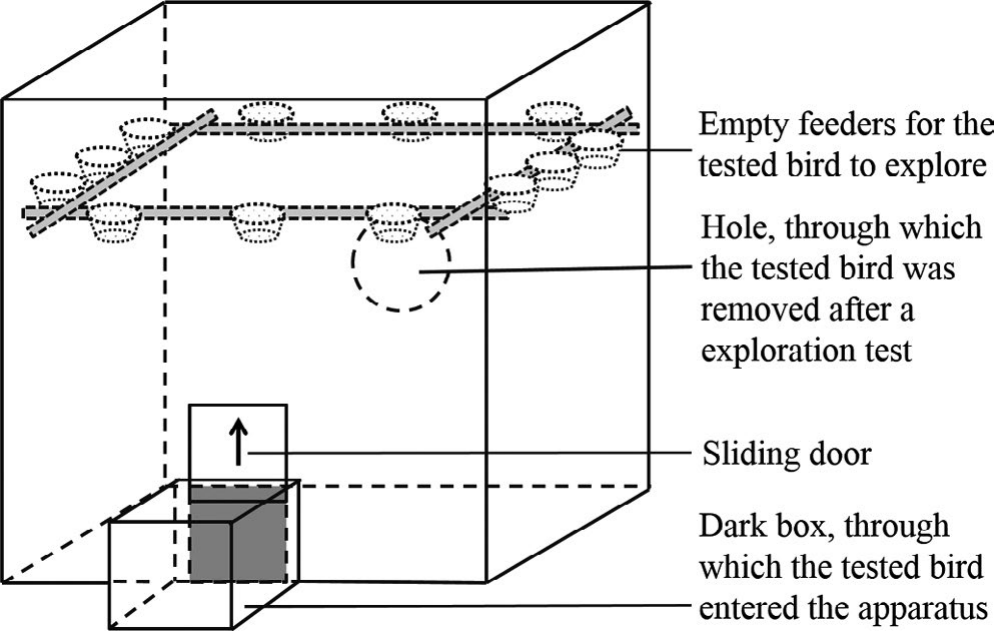
Schematic diagram of the apparatus for exploration and escape performance tests

Exploration tendency was assessed by introducing each individual into a cube‐shaped apparatus (50 cm × 50 cm × 50 cm; Figure 1). The apparatus had all walls opaque except the top, through which the behaviors of a bird could be filmed by a video camera. In the apparatus, 12 empty feeders were placed alongside the walls for the birds to explore. To motivate the birds' exploration, the feeders were the same type as those in their daily living cages. Also, each tested bird was deprived of food for 0.5 hr in an isolated small cage (27 cm × 13 cm × 20 cm) before being introduced into the experimental apparatus. After the deprivation of food, the tested bird was then transported to a dark box (14 cm long × 11 cm wide × 15 cm high) attached outside to the entrance of the exploration apparatus. A sliding door between the dark box and exploration apparatus was then removed, allowing the bird to enter the exploration apparatus. A video camera set above the apparatus recorded the process from the bird being placed in the dark box until its removal from the apparatus. During the 5 min after a bird entered the apparatus, the total number of visits to feeders (including repeated visits to the same feeder), the number of feeders visited (i.e., if the same feeder was visited twice or more, it was counted as one), and the total time that the bird stayed still (the opposite side of movement, reflecting its exploration activity) were employed to represent its exploration tendency.

All of the 30 birds were initially involved in the exploration test, but one adult escaped from the apparatus, reducing the sample size to 29 (26 first‐year individuals and three adults).

### 2.5 Test for escape performance

Escape performance was tested after each bird's exploration test. Through the hole on the sidewall of the exploration apparatus (covered by a sliding door during exploration tests; Figure 1), the bird was caught by an experimenter (J. Guo) with a scoop net. The catch‐escape process (recorded by the video camera) was used to evaluate the bird's ability to escape from potential predation. For this purpose, the time spent in catching each bird and the number of times that the bird escaped from the net (the birds often struggled to get out of the net once they were caught) were recorded. Although the birds usually kept hopping and flitting to different directions in the apparatus while being caught, the experimenter was able to execute proficient and constant skills to catch them both clockwise and anticlockwise after being trained on the six pilot birds mentioned above. Therefore, the right‐handedness of the experimenter was believed to not affect the time spent in catching differently footed birds. The sample size in the escape performance test is the same as that in exploration tendency tests.

### 2.6 Statistics

To access whether footedness was repeatable over time, we calculated the repeatability *R* (the fraction of the total variance attributed to variation among individuals) using the R package “rptR” (Nakagawa & Schielzeth, 2010; Stoffel, Nakagawa, & Schielzeth, 2017) in R v3.6.1 (R Development Core Team, 2019). The calculation was based on a generalized linear mixed model (GLMM) with a binomial distribution and a logit link using the “lme4” package (Bates, Maechler, Bolker, & Walker, 2015). In the GLMM, a bird's footedness, expressed as the proportion of right foot use among the all in each round of footedness test (range 0–1), was treated as the response variable, the round number of the footedness test as a controlling explanatory variable, and the bird ID as a random factor. The among‐individual variance was thus extracted from the variance of the random effect of bird ID. We calculated repeatability for LIFF, LILF, LITN, and LITT, respectively, and reported adjusted original‐scale repeatability (±*SE*).

Among the four footedness indexes, LITT was found to have the highest repeatability (see Results) and was chosen as the footedness index for the subsequent analyses. To report population‐level bias in footedness and analyze the relationship of footedness with other behavioral traits, we classified individuals as left‐footed if LITT ∈ [−1, −0.33), no preference if LITT ∈ [−0.33 to +0.33], or right‐footed if LITT ∈ (+0.33, +1]. For each individual that was involved in repeated footedness tests, an average LITT across the tests was calculated and was used for classifying it into one of the above three categories. The distribution of individuals among the three categories was analyzed with a chi‐square test.

To compare the feeding efficiency of differently footed individuals, we conducted a GLMM analysis treating the time that a bird spent in eating each mealworm in a footedness test as the response variable and the bird identity as a random factor to account for repeated tests on a bird. As the response variable had a right‐skewed distribution, the model was fitted using a Gamma distribution with a log link. The explanatory variables were the footedness (left‐footed, no preference, or right‐footed) and age (adult or young) of the bird. Because hunger level may affect the speed of eating and a hungry bird may eat the first few mealworms faster than eat later ones, the numerical order (i.e., 1st, 2nd, and 3rd) of the mealworm being eaten by a bird in the footedness test was also included as an explanatory model term. Denominator degrees of freedom of the GLMM analyses were obtained by Satterthwaite approximation as the data were unbalanced (Heck, Thomas, & Tab ata, 2012).

For the behavioral traits measured in the exploration and escape performance tests, the distribution of the data varied by the behavioral traits. So we analyzed different traits using different methods. The total number of visits to feeders followed a normal distribution (Kolmogorov–Smirnov test: *D* = 0.140, *df* = 29, *p* = .150), so the difference in this measure among differently footed birds was assessed by a one‐way ANOVA analysis. The distributions of three other measures including the time that a bird stayed still, the time to catch a bird, and the number of times that a bird escaped from the scoop net were all right‐skewed. Because the number of feeders visited during the exploration test showed a left‐skewed distribution, we converted it to the number of feeders that a bird had not visited by subtracting the number of feeders visited from 12 (the total number of feeders placed in the exploration apparatus). This latter new measure was thus right‐skewed distributed and reflected the coverage of the feeders in a bird's exploration activity. For these right‐skewed distributed measures, generalized linear models (GLMs) with a Gamma distribution and a log link were used to analyze their relationships with footedness. In each of the GLM analyses, the measure of a behavioral trait was treated as the response variable and the footedness as the explanatory variable. Because the number of feeders that a bird had not visited and the time that a bird stayed still during exploration test as well as the number of times that a bird escaped from the scoop net when being caught contained zero values, we added 0.1 to the original values of these measurements as GLMs with Gaussian distribution cannot handle data with zero. Adult and first‐year individuals did not significantly differ in all the measurements (Mann–Whitney *U* tests: all *p* > .05, see Table S2), except for the time spent for catching a bird after the exploration test (Mann–Whitney *U* tests: *U* = 6.500, *N_a_*_dult_ = 3, *N*_1st‐year_ = 26, *p* = .020). Therefore, all birds were analyzed together in the above analyses, except for the relationship of footedness with the time to catch a bird, of which we also reported the result after excluding the adults.

Except for the repeatability test, all other analyses were performed with IBM SPSS Statistics 25.0 (IBM). Tests were two‐tailed and were reported significant when *p* < .05.

## 3 RESULTS

### 3.1 Repeatability of footedness and population‐level bias

**Figure 2:**
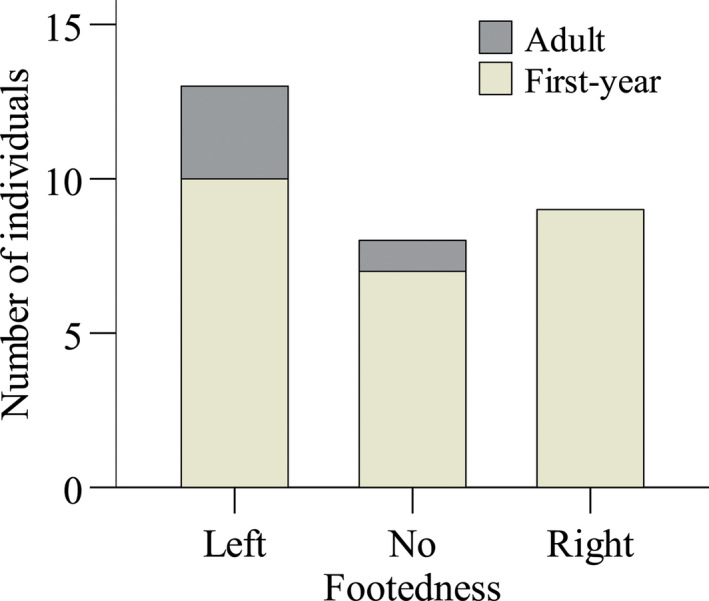
Distribution of footedness among the yellowed‐bellied tits in this study

Repeatability of different footedness indexes differed, with *R*_LIFF_ = 0.171 ± 0.060 (*p* < .001), *R*_LITN_ = 0.139 ± 0.049 (*p* < .001), *R*_LILF_ = 0.655 ± 0.230 (*p* < .001), and *R*_LITT_ = 0.971 ± 0.357 (*p* < .001). LITT, the footedness index based on the total time spent in using a given foot had the highest repeatability and was used as the footedness index to classify an individual into a footedness category. No population‐level bias in the three footedness categories was found among all the tested individuals (Chi‐square test: $\chi_{2}^{2}$ = 1.400, *p* = .497; Figure 2). The four adults were classified as one individual showing no preference for foot use and the other three having left footedness. After excluding the adults, there was also no population‐level bias in footedness among the first‐year individuals (chi‐square test: $\chi_{2}^{2}$ = 0.538, *p* = .764).

### 3.2 Correlation of footedness with other behaviors

**Figure 3:**
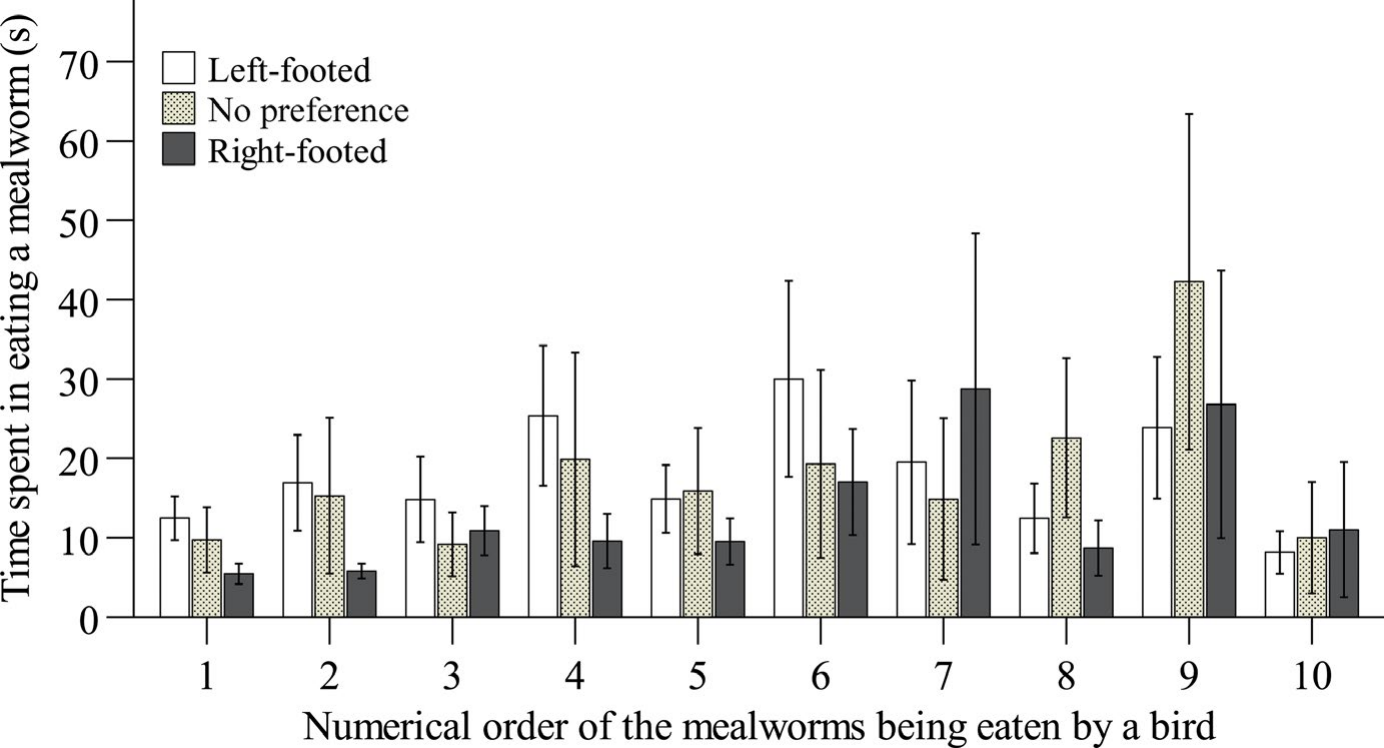
The relationship of the time (±*SE*) spent in eating a mealworm by a bird and the numerical order of the mealworm being eaten during the footedness test

The time that the yellowed‐bellied tits spent in eating a mealworm was not affected by either footedness (*F*_2,23_ = 1.169, *p* = .328) or age (*F*_1,24_ = 2.477, *p* = .129), but was significantly associated with numerical order of the mealworm that a bird ate during a footedness test (*F*_9,191_ = 2.514, *p* = .010), with a trend for the time to increase when more mealworms were eaten by a bird (Figure 3).

**Figure 4:**
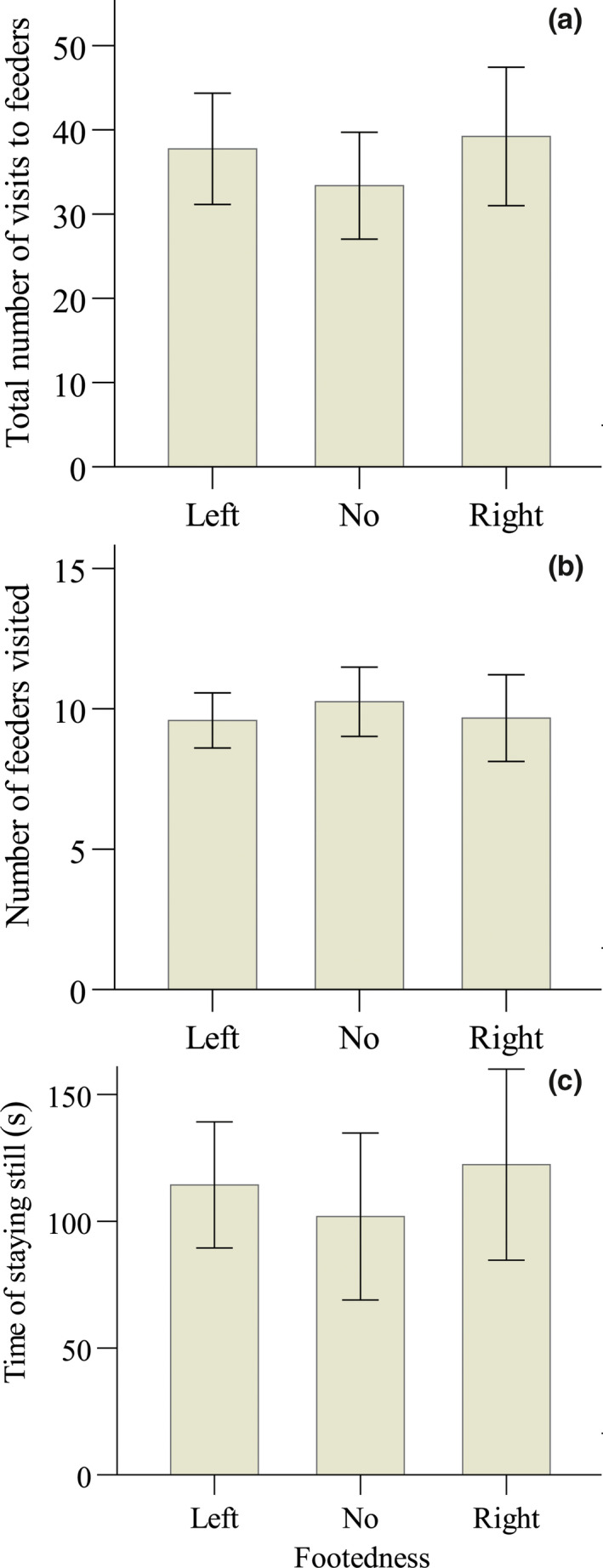
Comparisons of mean (±*SE*) exploration indexes between yellow‐bellied tits of different footedness: (a) total number of visits to feeders, (b) total number of feeders visited, and (3) time of staying still. Sample sizes for left‐footed, no preference, and right‐footed individuals were 12, 8, and 9, respectively. None of the indexes were significantly different between differently footed individuals

In the exploration test, individuals with different footedness also did not significantly differ in the total number of visits to feeders (one‐way ANOVA: *F*_2,28_ = 0.158, *p* = .855; Figure 4a), the number of feeders that a bird visited (GLM: $\chi_{2}^{2}$ = 207, *p* = .902; Figure 4b), or the time that a bird stayed still (GLM: $\chi_{2}^{2}$ = 0.124, *p* = .940; Figure 4c).

**Figure 5:**
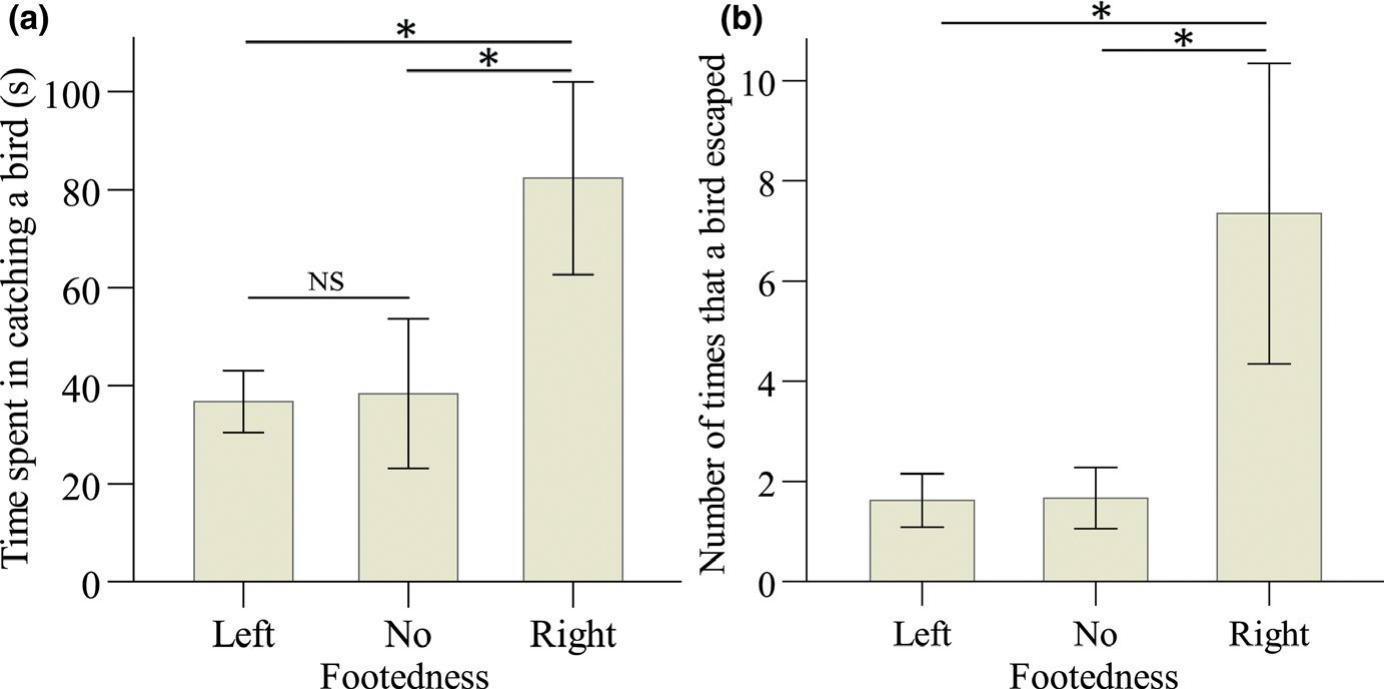
Comparisons of the mean (±*SE*) time spent catching a bird (a) and number of times that it escaped from the scoop net (b) between yellow‐bellied tits with different footedness. Sample sizes for left‐footed, no preference, and right‐footed individuals were 12, 8, and 9, respectively. *Significant (*p* < .05), whereas NS denotes a nonsignificant difference between differently footed individuals

After the exploration test, the time to catch a bird differed significantly among individuals of different footedness (GLM: all individuals, $\chi_{2}^{2}$ = 8.383, *p* = .015, Figure 5a; only first‐year individuals, $\chi_{2}^{2}$ = 6.807, *p* = .033). Compared to the right‐footed individuals, it took a shorter time to catch both the left‐footed (all individuals, estimated ± *SE* = −0.807 ± 0.300, $\chi_{1}^{2}$ = 7.237, *p* = .007; only 1‐year individuals, estimated ± *SE* = −0.700 ± 0.292, $\chi_{1}^{2}$ = 5.733, *p* = .017) and the no preference individuals (all individuals, estimated ± *SE* = −0.763 ± 0.330, $\chi_{1}^{2}$ = 5.338, *p* = .021; only first‐year individuals, estimated ± *SE* = −0.660 ± 0.321, $\chi_{1}^{2}$ = 4.236, *p* = .040).

The number of times that a bird escaped from the scoop net also varied significantly by their footedness (GLM: all individuals, $\chi_{2}^{2}$ = 10.156, *p* = .006; Figure 5b), with right‐footed individuals being more likely to escape than left‐footed (estimated ± *SE* = −1.514 ± 0.518, $\chi_{1}^{2}$ = 8.538, *p* = .003) and no preference individuals (estimated ± *SE* = −1.486 ± 0.571, $\chi_{1}^{2}$ = 6.774, *p* = .009).

## 4 DISCUSSION

We have shown that there are equivalent numbers of differently footed individuals among the tested yellow‐bellied tits and their footedness was repeatable. Moreover, the footedness was associated with a yellow‐bellied tit's escape performance but not feeding efficiency or exploration tendency.

A precondition for correlating behavioral laterality with other behavioral traits is that the lateralized behavior is stable during the observation time. The repeatability of behavioral laterality has been investigated in many vertebrates. For example, common marmosets have been found to show a preferred hand after the age of 5–12 months old and maintain the preference throughout their remaining life (Hook & Rogers, 2000). In wild chimpanzees (*Pan troglodytes*), the preference for using a certain hand remains stable after they are 10 years old (Boesch, 1991). In birds, an earlier study on hand‐reared great tits (*Parus major*) found that they start to use a specific foot for holding mealworms from about 10 days after fledgling and the preferred foot has been predominantly used in over 90% of occasions when they are 6 weeks after fledgling (Yince, 1964). Among the 30 yellow‐bellied tits used in the formal test of our study, 26 were born in the spring prior to being caught and the remaining four were at least at their second year of age. Since it was at least 4 months after these yellow‐bellied tits were born, their footedness may have been fully developed. Thus, it is reasonable for them to exhibit repeatability of footedness. Meanwhile, it should be acknowledged that whether their footedness will be stable in a longer period remains to be explored.

Our finding that the footedness of yellow‐bellied tits was repeatable makes it reasonable to further examine its relationship with other behaviors. In the test of the association of escape performance with footedness, we found the right‐footed individuals harder to catch and more likely to escape from the scoop net than both left‐footed and no preference individuals. This is, however, contrary to the prediction of left‐footed yellow‐bellied tits having better escape performance, but the following reasons may account for the right‐lateralized individuals' advantage in escape performance. On the one hand, while the right hemisphere is advantaged in response to risk, it is also known to be related to the expression of fear (Rogers, 2002, 2012; Rogers & Andrew, 2002). This could explain the relatively hypoactive performance of left‐footed individuals while escaping, because high levels of fear may result in behavioral disorder (e.g., panic disorder, Reiss, 1991). On the other hand, a brain with a hyperactive right hemisphere and a relatively hypoactive left hemisphere has been found to have indecisiveness problems (i.e., difficulty in making a decision) (Hecht, 2010), implying that left‐footed individuals may be disadvantaged in decision making. In accordance with this possibility, it has been found that the left‐handed marmosets are slower to emerge from a freeze response than right‐handed marmosets (Braccini & Caine, 2009). Thus, it is likely that although right hemisphere/left eye dominance may help to detect predators in some circumstances (Rogers, 2002; Rogers & Vallortigara, 2017), the right hemisphere dominance may also inhibit one's action because of fear, resulting in worse escape performance. To verify this possibility, future studies may need to investigate whether left‐footed yellow‐bellied tits prefer to use the left eye and perform better in detecting predators than right‐footed ones and if fear level, induced by predation stress, is correlated with escape performance.

We had expected that the right‐footed individuals, which may have a dominant left hemisphere, to have a higher feeding efficiency. However, our result does not support a relationship of a bird's footedness with the time of eating a mealworm. This may be because the advantage of left hemisphere/right eye dominance in feeding lies in helping an individual to ‘find’ foods, like the situations in chicks (Mench & Andrew, 1986), pigeons (Güntürkün & Kesch, 1987) and black‐winged stilts (Ventolini et al., 2005). However, the advantage of left hemisphere/right eye dominance does not necessarily correlate with the speed that an individual eat a food item, which is the situation in our study. Moreover, because our experimental design did not allow us to record yellow‐bellied tits' preferential eye use while feeding, it is unknown whether right‐footed individuals also preferred to use their right eye when feeding on mealworms. In this respect, it has been found in birds that parrots prefer to use their eye on the same side as the foot that they use to hold food items when feeding (Brown & Magat, 2011), but when searching for food, the chicks with an eye temporarily occluded by an eye patch tend to scratch the ground with the contralateral foot (Tommasi & Vallortigara, 1999). In addition, a few studies on other animals, such as common marmoset (Hook‐Costigan & Rogers, 1998) and chimpanzees (Braccini, Lambeth, Schapiro, & Fitch, 2012), reported no relationship between eye use and handedness. The mixed evidence suggests that handedness/footedness may be unrelated to eye use and thus does not necessarily predict feeding efficiency.

Exploration tendency is related to an individual's various aspects of life (Dingemanse & Réale, 2005; Réale et al., 2007), but lateralization in exploration has far been neglected as a field of investigation (Bonati & Csermely, 2013). We had expected left‐footed individuals to be either less explorative because of the correlation of right hemisphere dominance with the expression of fear, as being observed in the marmosets (Braccini & Caine, 2009), or more explorative for right hemisphere's advantage in processing spatial stimuli, as being observed in the common wall lizards (Bonati et al., 2013). However, not supporting either of the predictions, we detected no significant effects of footedness on the exploration indexes, including the number of visits to feeders, the number of feeders being visited, and the time that a bird stayed still. These results suggest that exploration may not relate to footedness in yellow‐bellied tits. Alternatively, it could be that the right hemisphere's advantage in processing spatial information may have been offset by the expression of fear, resulting in an ambiguous relationship between exploration and footedness. Apparently, the contrary predictions make the tests of exploration in relation to hemisphere dominance not straightforward, highlighting the need for future studies on their relationships to carefully tease apart the respective effects of the expression of fear and the processing of spatial information on exploring novel environments. Also, it is important to acknowledge that the apparatus for our exploration test was relatively small and simple, despite that we have attempted to use the feeders to attract the bird to explore. We are planning to employ a more rigorous experimental design to re‐examine the relationship between footedness and exploration behaviors in yellow‐bellied tits.

The knowledge of benefits and costs associated with behavioral laterality is a prerequisite for understanding why different biased individuals can coexist in a population. In juvenile Ambon damselfish (*Pomacentrus amboinensis*), Chivers et al. (2017) found the lateralized individuals to have stronger responses to the learned predator than nonlateralized ones, whereas they are poorer competitors, suggesting a balance of competing selection pressures. Our study found no population‐level bias in footedness in male yellow‐bellied tits. We had expected that differently footed yellow‐bellied tits may coexist if they have respective advantages under different circumstances. While we found the left‐ and right‐footed individuals performed equally well in feeding efficiency and exploration tendency, the finding that right‐footed yellow‐bellied tits are advantaged in escaping from potential predation raises a questions of why there are not more right‐footed individuals in the population, warranting future studies to investigate whether left footedness in yellow‐bellied tits is associated with other benefits.

The last point needed to be noted in our study is that the tests were conducted only on male yellow‐bellied tits. It has been reported that the extent of preferential use of one limb over the other, such as the paw use in domestic cat (*Felis silvestris catus*; Wells & Millsopp, 2009) and the handedness in chimpanzees (Hopkins, Russell, Schaeffer, Gardner, & Schapiro, 2009), may differ between females and males. Further studies on footedness of the female yellow‐bellied tits are therefore warranted.

To summarize, our study shows the footedness of yellow‐bellied tits is repeatable and the results do not support the predicted relationships of footedness with feeding efficiency and exploration tendency based on the role of dominant hemisphere corresponding to footedness. Nevertheless, this study provides the first evidence in birds that footedness is related to escape performance. We reiterate that limb use in birds can be suitable subjects for studies of behavioral laterality and more researches regarding the benefits/costs associated with behavioral laterality should be advocated for a better understanding of the mechanisms maintaining its diversity.

## CONFLICT OF INTEREST

The authors declare no conflict of interest.

## AUTHOR CONTRIBUTIONS

GY, JG, WX, JW, and YW designed and conducted experiments, and assisted in data analysis; JL conceived the study, supervised in designing the experiments and analyzed data; GY, JX, and JL wrote the manuscript; JZ contributed to pilot studies.

## ETHICAL APPROVAL

Our experiments are in line with the ASAB/ABS Guidelines for the Use of Animals in Research. The experimental procedures were approved by Ethical Committee of Animal Research of the School of Nature Conservation at Beijing Forestry University. The birds used in the study were captured by mist‐netting after breeding season with permits from Beijing Gardening and Greening Bureau (permit no. 2015‐160).

## Supporting information

Table S1‐S2

## Data Availability

Supportive data are available at Figshare (https://doi.org/10.6084/m9.figshare.11888718).
