# Supplementary material for: Footedness predicts escape performance in a passerine bird
Source: Ecol Evol. 2020 Apr 16;10(10):4251–60. doi: 10.1002/ece3.6193 (PMC7246196; doi:10.1002/ece3.6193)
Supplement: Supplementary file 1 — Table S1‐S2 [file ECE3-10-4251-s001.docx]

**Supplementary materials**

Table S1. Spearman correlations between different laterality indices of footedness in different repeats of tests

| Repeats | LI | LILF |  | LITN |  | LITT |  |
| --- | --- | --- | --- | --- | --- | --- | --- |
|  |  | N | r | N | r | N | r |
| The 1^st^ day | LIFF | 14 | 0.851^**^ | 14 | 0.955^**^ | 14 | 0.862^**^ |
|  | LILF |  |  | 14 | 0.942^**^ | 14 | 0.897^**^ |
|  | LITN |  |  |  |  | 14 | 0.912^**^ |
| The 4^th^ day | LIFF | 19 | 0.881^**^ | 19 | 0.910^**^ | 19 | 0.770^**^ |
|  | LILF |  |  | 19 | 0.966^**^ | 19 | 0.817^**^ |
|  | LITN |  |  |  |  | 19 | 0.830^**^ |
| The 7^th^ day | LIFF | 19 | 0.696^**^ | 19 | 0.834^**^ | 19 | 0.761^**^ |
|  | LILF |  |  | 19 | 0.955^**^ | 19 | 0.923^**^ |
|  | LITN |  |  |  |  | 19 | 0.947^**^ |
| The 14^th^ day | LIFF | 14 | 0.845^**^ | 14 | 0.834^**^ | 14 | 0.946^**^ |
|  | LILF |  |  | 14 | 0.739^**^ | 14 | 0.928^**^ |
|  | LITN |  |  |  |  | 14 | 0.851^**^ |
| The 21^st^ day | LIFF | 15 | 0.744^**^ | 15 | 0.943^**^ | 15 | 0.834^**^ |
|  | LILF |  |  | 15 | 0.860^**^ | 15 | 0.945^**^ |
|  | LITN |  |  |  |  | 15 | 0.935^**^ |
| The 28^th^ day | LIFF | 19 | 0.661^**^ | 19 | 0.895^**^ | 19 | 0.759^**^ |
|  | LILF |  |  | 19 | 0.891^**^ | 19 | 0.927^**^ |
|  | LITN |  |  |  |  | 19 | 0.909^**^ |

Notes: LIFF reflects the tendency of using a given foot to grasp food for the first time by a bird; LILF reflects the tendency of using a given foot to clamp the Mealworm against the perch when a bird was finishing eating each Mealworm; LITT reflects a bird’s preference for using a given foot to clamp the Mealworm against the perch during the whole process; and LITN is similar to LITT, but is calculated with the total number of times of using a foot, instead of the total time, during the whole process. *, P < 0.05；**, P < 0.01

Table S2. The Mann-Whitney tests on effect of age on exploration tendency and escape performance measurements

| Measurements | | N | | | | Mann-Whitney U | P |
| --- | --- | --- | --- | --- | --- | --- | --- |
|  |  | adult | | first-year | |  |  |
| exploration tendency | Total visit | | 3 | | 26 | 25.000 | 0.315 |
|  | Number | | 3 | | 26 | 28.500 | 0.400 |
|  | Stay still time | | 3 | | 26 | 34.000 | 0.720 |
| escape performance | Escape time | | 3 | | 26 | 14.000 | 0.068 |

Notes: Total visit is the total number of visits to feeders; Number is the number of feeders visited; Stay still time is the total time that the bird stayed still; Escape times is the number of times that the bird escaped from the net.
